# Supplementary material for: EnzML: multi-label prediction of enzyme classes using InterPro signatures
Source: BMC Bioinformatics. 2012 Apr 25;13:61. doi: 10.1186/1471-2105-13-61 (PMC3483700; doi:10.1186/1471-2105-13-61)
Supplement: Addtional file 5 — The Java code to format the data files, evaluate and predict. The file enzml_java_code.tar.gz contains the Java code used to format database data to ARFF and XML formats, to execute cross and train-test (jackknife) evaluations and to record evaluation results to database. More information is included in the readme.txt file and the Javadoc files. The code can be used with a MySQL database. To use a different database software, other JDBC drivers might be required. [file 1471-2105-13-61-S5.gz › java_code/enzml2011/doc/uk/ac/ed/inf/taweka/mulan/learn/package-use.html]

Uses of Package uk.ac.ed.inf.taweka.mulan.learn


---


|  |  |  |
| --- | --- | --- |
| **Overview** \| **Package** \| Class \| **Use** \| **Tree** \| **Deprecated** \| **Index** \| **Help** | |  |
| PREV   NEXT | **FRAMES**    **NO FRAMES**     **All Classes** |  |


---


## **Uses of Package uk.ac.ed.inf.taweka.mulan.learn**

| Packages that use uk.ac.ed.inf.taweka.mulan.learn | |
| --- | --- |
| **uk.ac.ed.inf.taweka.mulan.learn** |  |
| **uk.ac.ed.inf.taweka.mulan.predict** |  |
| **uk.ac.ed.inf.taweka.test.mulan.learn** |  |

Classes in uk.ac.ed.inf.taweka.mulan.learn used by uk.ac.ed.inf.taweka.mulan.learn

****MulanArffRecord****
  
          Checks that an ARFF record read from the database contains valid parameters
to read an ARFF file.

****MulanCrossEvaluator****
  
          Class created to execute cross-evaluations and extract individual evaluation
results (not possible from the Mulan class Evaluator, because only averaged
results are returned).

****MulanCrossExperimenter****
  
          Using Mulan (multi-label machine learning), this class initialises and
executes either Train-Test evaluation (if two different arff records are
given) (calling `MulanTrainTestEvaluator`) or cross-evaluations (calling
`MulanCrossEvaluator`) if only one arff record is given.

****MulanLearner****
  
          Containing a Mulan learner and its name, that is: the name of the
MultiLabelLearnerBase plus the name of the algorithm within

****ResultsSaver****
  
          Outputs cross-evaluation results to screen, file and database.

****TrainTestEvaluator****
  
          Train/test evaluator.

****TrainTestExperimenter****
  
          Using Mulan (multi-label machine learning), this class initialises and
executes Train-Test evaluation (two arff records are needed, for the training
and testing files) (calling `MulanTrainTestEvaluator`).

Classes in uk.ac.ed.inf.taweka.mulan.learn used by uk.ac.ed.inf.taweka.mulan.predict

****MulanArffRecord****
  
          Checks that an ARFF record read from the database contains valid parameters
to read an ARFF file.

Classes in uk.ac.ed.inf.taweka.mulan.learn used by uk.ac.ed.inf.taweka.test.mulan.learn

****MulanCrossEvaluator****
  
          Class created to execute cross-evaluations and extract individual evaluation
results (not possible from the Mulan class Evaluator, because only averaged
results are returned).

****MulanCrossExperimenter****
  
          Using Mulan (multi-label machine learning), this class initialises and
executes either Train-Test evaluation (if two different arff records are
given) (calling `MulanTrainTestEvaluator`) or cross-evaluations (calling
`MulanCrossEvaluator`) if only one arff record is given.

****MulanLearner****
  
          Containing a Mulan learner and its name, that is: the name of the
MultiLabelLearnerBase plus the name of the algorithm within

****MulanSerializer****
  
          Takes an arff file and an algorithm.

****ResultsSaver****
  
          Outputs cross-evaluation results to screen, file and database.

****TrainTestEvaluator****
  
          Train/test evaluator.

****TrainTestEvaluatorSerialised****
  
          Class

****TrainTestExperimenter****
  
          Using Mulan (multi-label machine learning), this class initialises and
executes Train-Test evaluation (two arff records are needed, for the training
and testing files) (calling `MulanTrainTestEvaluator`).

****TrainTestExperimenterSerialized****
  
          Executes a train/test evaluation using a previously trained model saved on
file.

---


|  |  |  |
| --- | --- | --- |
| **Overview** \| **Package** \| Class \| **Use** \| **Tree** \| **Deprecated** \| **Index** \| **Help** | |  |
| PREV   NEXT | **FRAMES**    **NO FRAMES**     **All Classes** |  |


---
